# Expression of MYSM1 is associated with tumor progression in colorectal cancer

**DOI:** 10.1371/journal.pone.0177235

**Published:** 2017-05-12

**Authors:** Yongmin Li, Jingwen Li, He Liu, Yanlong Liu, Binbin Cui

**Affiliations:** Department of Colorectal Surgery, The Affiliated Tumor Hospital of Harbin Medical University, Harbin, China; Sapporo Ika Daigaku, JAPAN

## Abstract

Colorectal cancer, the third most common cancer in both men and women, has gradually increased in recent years. MYSM1has been investigated as a regulator of hematopoiesis and lymphocyte development in human. It has been reported that some tumor-related genes were modulated by MYSM1. However, its exact role in cancer development remains unclear. Herein, we aimed to examine the expression level of MYSM1 in tumor tissues and its correlation with clinicopathology and survivals of patients with colorectal cancer (CRC).MYSM1expressions in tumor specimens resected from 123 CRC patients were detected by immunochemistry and Western blot analysis. The results showed that MYSM1 was significantly highly expressed in carcinoma tissues compared with adjacent normal mucosa tissues (P<0.05). Correlation analyses by Pearson’s chi-square test demonstrated that MYSM1 in tumors was positively correlated with tumor status (pathological assessment of the primary tumor (pT, P<0.001), regional lymph nodes (pN, P = 0.013), distant metastasis (pM, P<0.001)) and clinic stage (P<0.001); Whereas, MYSM1 was not associated with tumor size of CRC patients and was positively associated with tumor differentiation grade (P = 0.015). Patients with positiveMYSM1expression showed poor survival compared with the MYSM1 negative group (P<0.001).Simultaneously, multivariate Cox regression analysis indicated thatMYSM1 expression in tumor cells was an independent factor for reduced overall survival in CRC patients (P<0.001).Additionally,MYSM1 in CRC SW480 cells was silenced by small interference RNA (siRNA) technology. Scratch assay and Transwell assay showed that MYSM1 silencing decreased migration and invasion abilities of SW480 cells. These data suggested that expression of MYSM1 was associated with the progression of CRC and might be a potential biomarker for clinical prognosis.

## Introduction

The prevalence of colorectal cancer (CRC), one of the most common cancers, has gradually increased in recent years. Various of biomarkers that correlate with clinical significances have been explored in scientific research or used in clinical practice, however, with it slimited prognostic power using biomarkers in clinical practice. Inconvenience of endoscopy, the golden criteria of CRC diagnosis, and the lack of prognostic marker lead to late diagnosis and treatment of malignancy, distant metastasis and consequently low 5-year survival rates.

Myb-like SWIRM and MPN domains 1 (MYSM1) is a chromatin-binding transcriptional cofactor that deubiquitinates histone H2A lysine 119 with mono-ubiquitinylation[1]. MYSM1 is an epigenetic regulator essential for the maintenance of hematopoietic stem cell (HSC) function, hematopoietic progenitor survival, and lymphocyte development. The loss of MYSM1 in mice contributes to severe hematopoietic dysfunction, the arrest of lymphocyte development and impaired multipotent progenitors (MPPs) survival[2–4].MYSM1 acts by coordinating histone acetylation and deubiquitination, and destabilizing the association of linker histone H1 with nucleosomes [1]. Additionally, MYSM1 participates in transcriptional regulation events in androgen receptor-dependent gene activation. It has been found that MYSM1-deficience-triggered impairment in hematopoieticstem cell (HSC) function is associated with the elevated tumor suppressor protein p53 expression [2,4]. It has also been reported that p53 is activated in MYSM1-deficient mice and stimulates the upregulation of p53-induced mediators of apoptosis, leading to hematopoietic defects and cell cycle arrest [5]. p53, as a DNA-binding protein, is central regulator of cell fate and cellular stress responses [6]. It functions by regulating transcription to trigger cell cycle arrest, cell aging and apoptosis [7]. Therefore, we speculated that MYSM1 might play an important role in cancer progression and development. Moreover, levels of monoubiquitinylated H2A are dramatically decreased in prostate tumors and MYSM1 is required for the activation of several target genes in prostate cancer cells [1], suggesting MYSM1 expression may serve as a cancer marker. However, the clinical significance of MYSM1 in CRC has not yet been reported.

In this study, we examined the expression level of MYSM1in 221 resected specimens from 123 CRC patients by immunohistochmeistry staining and explored their association with clinicopathology and prognostic factors of CRC, and thus determined the clinical relevance of MYSM1 expression in distinct CRC types.

## Methods

### Patient and tissue specimens

The 221 formalin-fixed paraffin-embedded tissue samples used to IHC were collected from 123 CRC patients undergoing surgery in 2005–2010, and were grouped as tumor tissue adjacent normal mucosa (n = 30), primary carcinoma (n = 123), lymphnode metastasis (n = 30) and liver metastasis tissue(n = 38). And all of the the normal mucosa were collected from the 123 CRC patients. Similarly, there were matching lymphnode metastasis tissues for 30 primary carcinomas and matching liver metastasis tissue for 38 primary carcinomas. Primary carcinomas were assessed according to the 7th edition American Joint Committee on Cancer (AJCC) staging system. All data and tissues of CRC patients as paraffin blocks were collected from the Affiliated Tumor Hospital of Harbin Medical University. The patients had a median age of 60 years (range, 35 to 90 years); 73 (59.3%) were males and 50 (40.7%) were females.There were 54 (43.9%) cases of Stage I and II tumors and 69 (56.1%) cases of Stage III and IV tumors based on the International Union against Cancer 2002 TNM staging system and WHO classification criteria [8].The median follow-up period was 23.1 months (range, 1–106 months).No patients received the preoperative radiotherapy or chemotherapy. The study was approved by the Ethics Committee of the Affiliated Tumor Hospital of Harbin Medical University, Harbin, China. And all of the donors or the next of kins had written the informed consent for use of the tissue sample in research.

### Cell line and RNA interference

The human CRC cell line SW480 was obtained from the Cancer Research Institute of Harbin Medical University. The cell lines were maintained in RPMI-1640(Gibco-BRL, Carlsbad, CA, USA) supplemented with 10% fetal bovine serum (ExCell Bio, Shanghai, China), 100 μg/ml streptomycin and 100 units/ml penicillin (Invitrogen, Carlsbad, CA, USA)and cultured at 37°C in a humidified atmosphere containing 5% CO2.

siRNA sequence for *MYSM1* and the control siRNAwere designed using Ambion software, and were synthesized and purchased from Invitrogen(*MYSM1* siRNA: Sense 5’-UCUGGCCGGUAUUAGAAGUUCAAUU-3’, Antisense 5’-AAUUGAACUUCUAAUACCGGCCAGA-3’; Negative control siRNA: Sense 5’-UUCUCCGAACGUGUCACGUTT-3’, Antisense 5’-ACGUGACACGUUCGGAGAATT-3’). Cells were seeded in 6-well plates (at 40 to 50% confluence) and starved for 4 h. siRNA transfection was performed with X-treme GENE transfection reagent (Roche, NJ, USA) according to the manufacturer’s instructions. The CRC cells in each group were transfected for further research needs by siRNAs for at least 48h.

### Western blot analysis

Cells were washed twice using cold PBS, and then lysed in RIPA cell lysis buffer (Beyotime Biotechnology, Haimen, China) containing a protease inhibitor cocktail (Roche, NJ, USA) at 4°C for 15 min. Following centrifugation at 12,000 x g for 10 min, the supernatant was collected and quantified using a bicinchoninic acid quantification kit (Beyotime Biotechnology). The proteins (30 μg) were separated by 10% SDS-PAGE (Beijing Solarbio Science & Technology Co., Ltd.)and transferred to polyvinylidene fluoride (PVDF) membranes (Millipore, MA, USA). The membranes were blocked with 5% non-fat dried milk in TBST for 1 h, and incubated overnight at 4°C with specific primary antibodies: Rabbit polyclonalMYSM1 antibody (1:500, ab180570, Abcam, Cambridge, USA) and mouse monoclonal β-actin antibody (1:1000, Zhongshanjinqiao Bio-Technology Co. Ltd., Beijing, China). Horseradish peroxidase (HRP)-conjugated secondary antibodies: goat anti-mouse (1:2000; cat. no. sc-2005,Santa Cruz, CA, USA) and goat anti-rabbit IgG (1:2000; cat. no. sc-2004,Santa Cruz, CA, USA) were used for incubation at room temperature for 2 h. Development was performed using ECL-detecting reagent (Tanon, Shanghai, China)[9].

### Immunohistochemistry (IHC)

The normal mucosa and primary carcinoma tissues used for IHC were obtained from the same patient. The formalin-fixed, paraffin-embedded sections (4μm) were deparaffinized, rehydrated, and then quenched with 3% H_2_O_2_ for 10 min. Antigen retrieval were performed by autoclave in 10 mM sodium citrate buffer (pH6.0) for 3 min at 121°C. The sections were then immersed in a 0.3% hydrogen peroxidesolution for 30 min to block endogenous peroxidase activity.After washing with PBS for 5 min, the sections were incubated with MYSM1 antibody (diluted at 1:200) overnight at 4°C. A negative control was performed by replacing the primary antibody with a normal rabbit IgG antibody, indicating the specifity of antibody. Then the sections were incubated with HRP-conjugated mouse anti-rabbitsecondary antibody (1:1000, PV6001, Zhongshan Goldbridge Biotechnology, China) for 20 min. 3, 3′-diaminobenzidine tetrahydrochloride (DAB) was used for development and the slides were counterstained using Mayer’s hematoxylin.

Two experienced pathologists blinded to the clinicopathological information scored the MYSM1 level in tumor cells by assessing (a) the proportion of positively stained cells: (0, <5%; 1, 6 to 25%; 2, 26 to 50%; 3, 51 to 75%; 4, >75%) and (b) the signal intensity: (0, no signal; 1, weak; 2, moderate; 3, strong). The score was determined by multiplying a and b[10].The level of MYSM1 expression was obtained by counting the positively and negatively stained cells in five to ten separate 400× high-power microscopic fields and calculating the mean percentage of positively stained cells among the total cells per field.

### Scratch assay

A wound-healing assay was performed to detect cell migration. The prepared cells were seeded in 6-well plates, and after the cells reached 90–100% confluence, a 10-μl sterile micropipettor tip was used to create a void of cells by scratching. The plates were then washed to remove the dislodged cells. The CRC cells were cultured in medium containing 5% FBS. The initial gap length (0 h) and the residual gap length at 48 h after wounding were calculated from photomicrographs[11].

### Transwell assay

Cell invasion was evaluated by Transwell assay in 24-well transwell plates (8 μm pore size, Corning Costar, USA)[12]. The chamber inserts were coated with Matrigel membrane (BD Biosciences, USA). RPMI-1640 containing 20% fetal bovine serum in the lower chamber served as the chemoattractant. Cells (5×10^5^/well) were incubated at 37°C for 48 h.The cells on the undersurface of the upper chamber were presented by crystal violet (Amresco, USA) staining for 10 minutes at room temperature, and then were observed using a light microscope (Olympus, Japan) and chose six random fields at 100× magnification to score the average cell coverage.

### Statistical analysis

The IBM SPSS Statistics version 21.0 (IBM Co., Armonk, NY, USA) software was used for the statistical analysis. A chi-square test was used to analyze the relationship between the expression of MYSM1 and various clinicopathologic parameters. Overall survival was evaluated to determine the prognostic value of MYSM1 expression using the Kaplan-Meier method. P<0.05 was considered statistically significant. Data was presented as the mean ± standard error of the mean (SEM).

## Results

### Expression of MYSM1 in CRC

**Fig 1:**
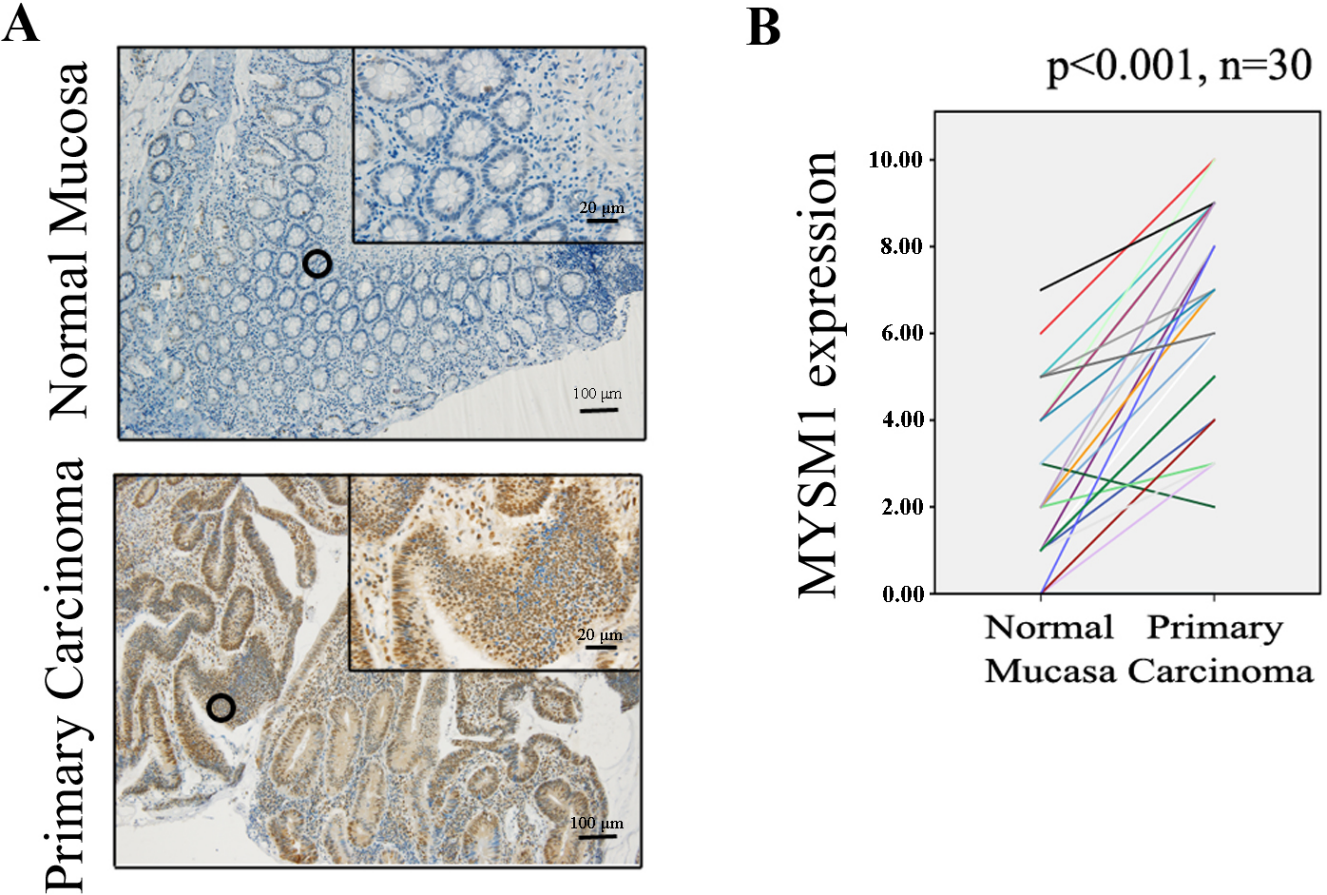
Immunohistochemical staining for MYSM1 in human colorectal tissues. (A) Expression levels of MYSM1 in normal mucosa (n = 30) and primary carcinoma tissues (n = 30)from patients with CRC were detected by immunohistochemical staining (×10, 100 μm; ×40, 20 μm).(B)MYSM1 protein levels in normal mucosa and primary carcinoma tissues were quantified as shown as line chart.

**Fig 2:**
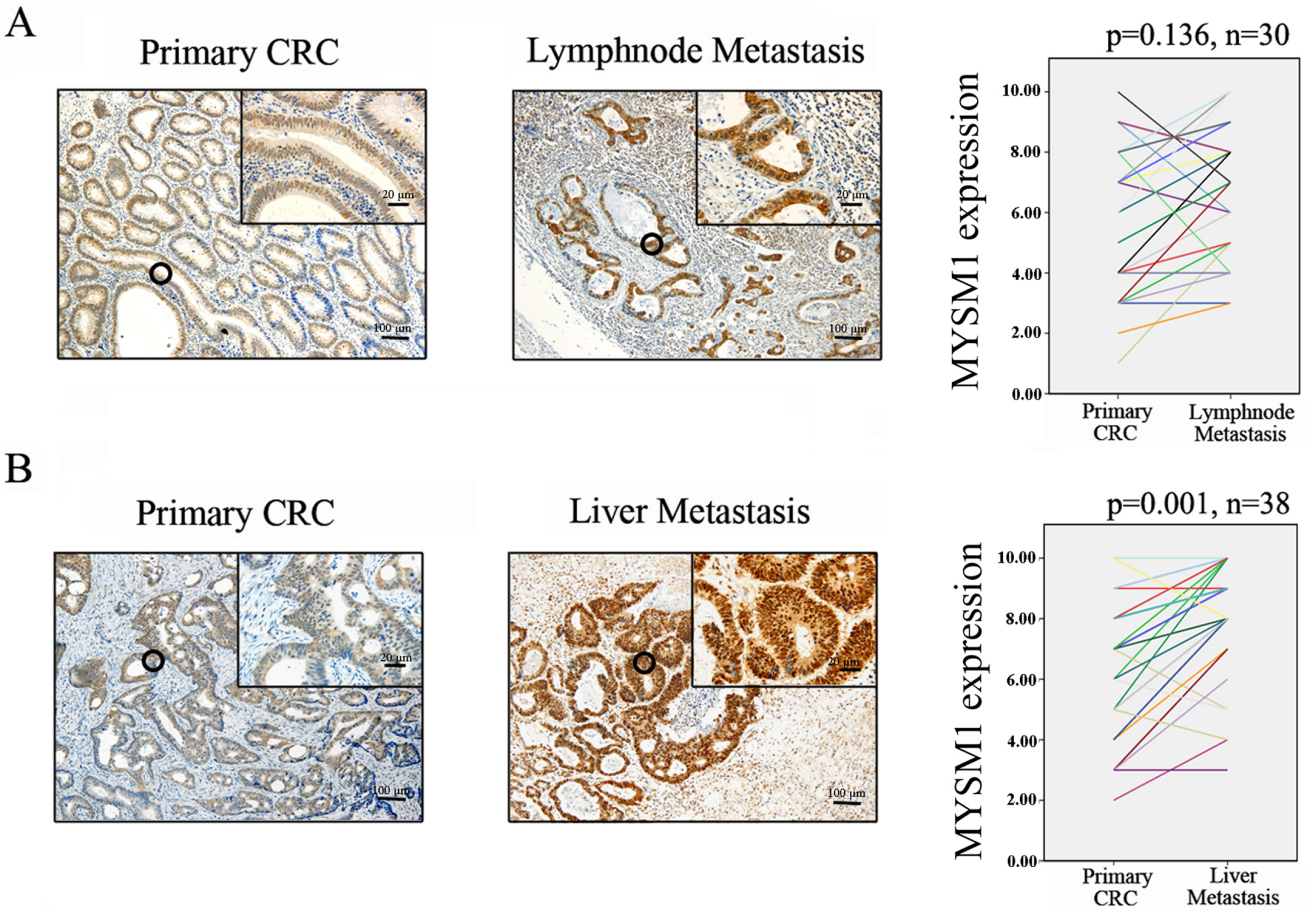
Immunohistochemical staining for MYSM1 in human CRC tissues. (A) Expression levels of MYSM1 in primary carcinoma (n = 30) and lymphnode metastasis tissues (n = 30)were detected (×10, 100 μm; ×40, 20 μm). (B)Expression levels of MYSM1 in primary carcinoma (n = 38) and liver metastasis tissues (n = 38) were examined (×10, 100 μm; ×40, 20 μm).

**Fig 3:**
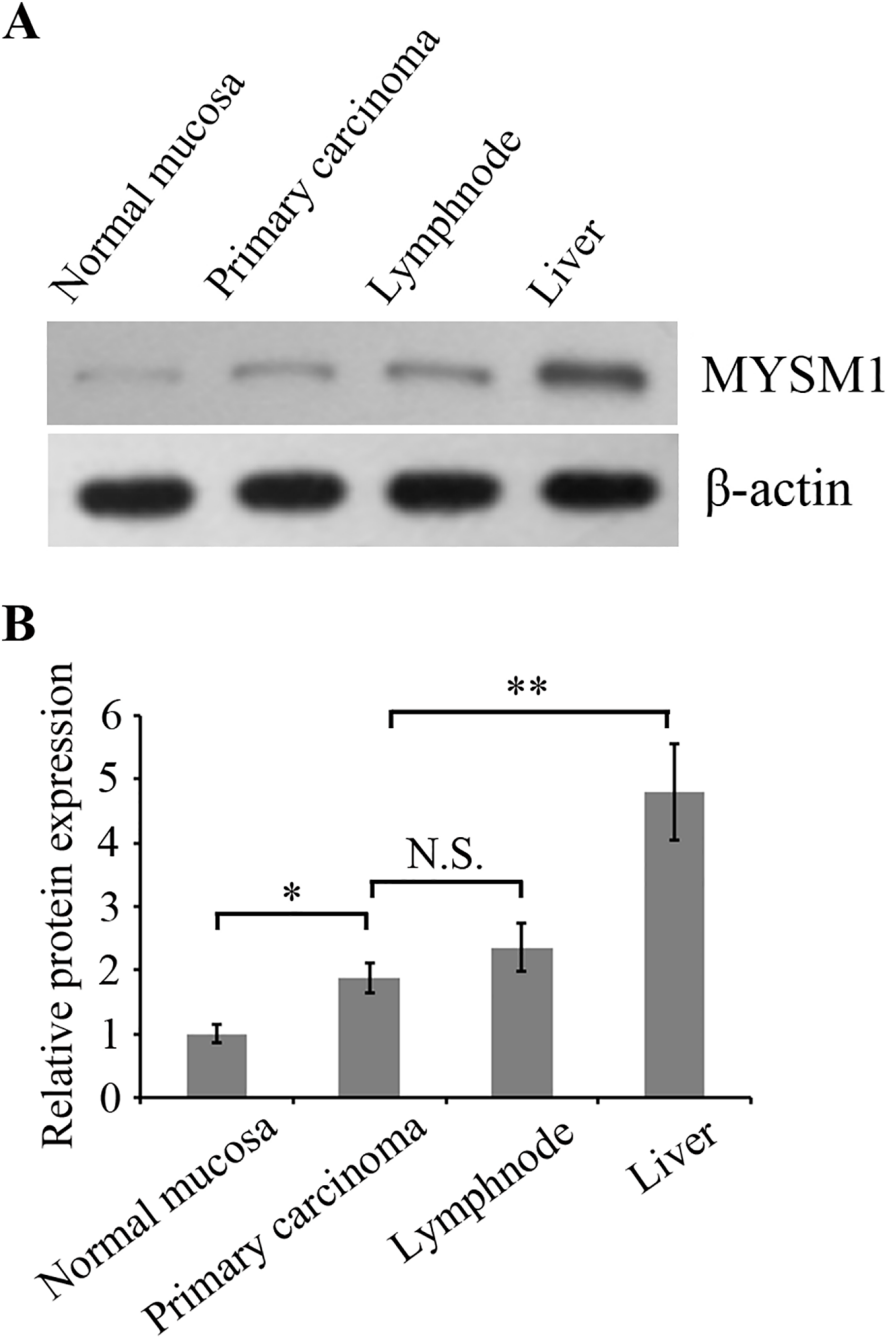
Western blot analysis for MYSM1 in human CRC tissues. (A) Expression levels of MYSM1 in normal mucosa, primary carcinoma, lymphnode metastasis tissues and liver metastasis tissues were examined by Western blot using the corresponding antibody.β-actin was detected as internal reference.(B) The bar graph showed the amount of interest proteins normalized to the amount of β-actin.*P < 0.05compared with normal mucosa, **P < 0.01 compared with primary carcinoma, mean ± SEM. N.S., no significance.

Protein levels of MYSM1 in normal mucosa (n = 30) and primary carcinoma (n = 30) tissues from CRC patients were detected by immunohistochemical staining. The results showed that MYSM1 (P<0.001)was significantly highly expressed in primary carcinoma tissues (protein level:moderate) compared with normal mucosa tissues(protein level: weak) (Fig 1A and 1B). Protein level of MYSM1 in primary carcinoma (n = 30) and lymphnode metastasis tissues (n = 30) detection indicated that MYSM1 protein expression in lymphnode metastasis tissues did not exhibit significant elevation relative to primary carcinoma groups (P = 0.136) (Fig 2A). Comparison between protein level of MYSM1 in primary carcinoma (n = 38) and that in liver metastasis tissues (n = 38) showed that MYSM1 (P = 0.001) was significantly increased in liver metastasis tissues (protein level: strong) compared with primary carcinoma tissues (Fig 2B). Western blot analysis was used to detect MYSM1 protein levels in patient tissues and showed the identical results to immunohistochemical staining (Fig 3A and 3B).These results suggest that MYSM1 is involved in tumorigenesis and metastasis of CRC. In brief, MYSM1 is positively associated with tumorigenesis and liver metastasis of tumor cells.

### MYSM1 correlates with clinicopathologic features

The correlations between MYSM1 and clinicopathologic features in tissue samples from 123 CRC patients were demonstrated in Table 1. The patients were divided into two groups(a MYSM1-positive group and a MYSM1-negative group) based on the medians of immunohistochemical variable values in diverse cell subsets.MYSM1 expression in tumors was shown to be positively correlated with tumor status (pathological assessment of the primary tumor (pT, P<0.001); regional lymph nodes (pN, P<0.001); distant metastasis (pM, P<0.001), clinic stage (P<0.001) and tumor differentiation grade (P = 0.015). Whereas, MYSM1 expression was not associated with tumor size of CRC patients. In addition, the tumor marker CA199 was also shown to be positively associated with MYSM1 positive expression (P = 0.007), suggesting that MYSM1 expression in tumor cells is a potential predictive factor for CRC.

**Table 1:** MYSM1 staining in tumor cells and associations with clinicopathologic characteristics.

Clinicopathologic parameter	MYSM1				
	No.	Negative (%)	Positive (%)	X^2^	P
**Age**				0.035	0.852
<60	68	31 (45.6)	37 (54.4)		
≥60	55	26 (47.3)	29 (52.7)		
**Gender**				0.453	0.501
Male	73	32 (43.8)	41 (56.2)		
Female	50	25 (50.0)	25 (50.0)		
**Location**				2.357	0.125
Colon	50	19 (38.0)	31 (62.0)		
Rectum	73	38 (52.1)	35 (47.9)		
**Tumor size(cm)**				0.926	0.336
<5	59	30 (50.8)	29 (49.2)		
≥5	64	27 (42.2)	37 (57.8)		
**Differentiation**				5.929	0.015*
Well/Moderate	72	40 (55.6)	32 (44.4)		
Poor/Mucinous	51	17 (33.3)	34 (66.7)		
**pT classification^a^**				17.397	<0.001*
T1-T3	49	34 (69.4)	15 (30.6)		
T4	74	23 (31.1)	52 (68.9)		
**pN classification^b^**				6.207	0.013*
N0	65	37 (56.9)	28 (43.1)		
N1-N2	58	20 (34.5)	38 (65.5)		
**pM classification^c^**				20.641	<0.001*
M0	85	51 (60.0)	34 (40.0)		
M1	38	6 (15.8)	32 (84.2)		
**AJCC stage^d^**				26.598	<0.001*
I	25	20 (80.0)	5 (20.0)		
II	29	14 (48.3)	15 (51.7)		
III	31	17 (54.8)	14 (45.2)		
IV	38	6 (15.8)	32 (84.2)		
**CEA**				3.63	0.057
≤5.0 (Negative)	62	34 (54.8)	28 (45.2)		
>5.0(Positive)	61	23 (37.7)	38 (62.3)		
**CA199**				7.176	0.007*
≤37.0 (Negative)	97	51 (52.6)	46 (47.4)		
>37.0(Positive)	26	6 (23.1)	20 (76.9)		
**Total**	123	57 (46.3)	66 (53.7)		

*P < 0.05, statistically significant, Pearson’s X^2^ test.Basis age cutoff was 60 years, tumor size cutoff was 5 cm, CEA cutoff was 5, and CA199 cutoff was 37.

^a^pathological assessment of the primary tumor

^b^pathological assessment of the regional lymph nodes

^c^pathological assessment of the distant metastasis

^d^American Joint Committee on Cancer.

### Correlation of MYSM1 expression with prognosis in CRC

**Fig 4:**
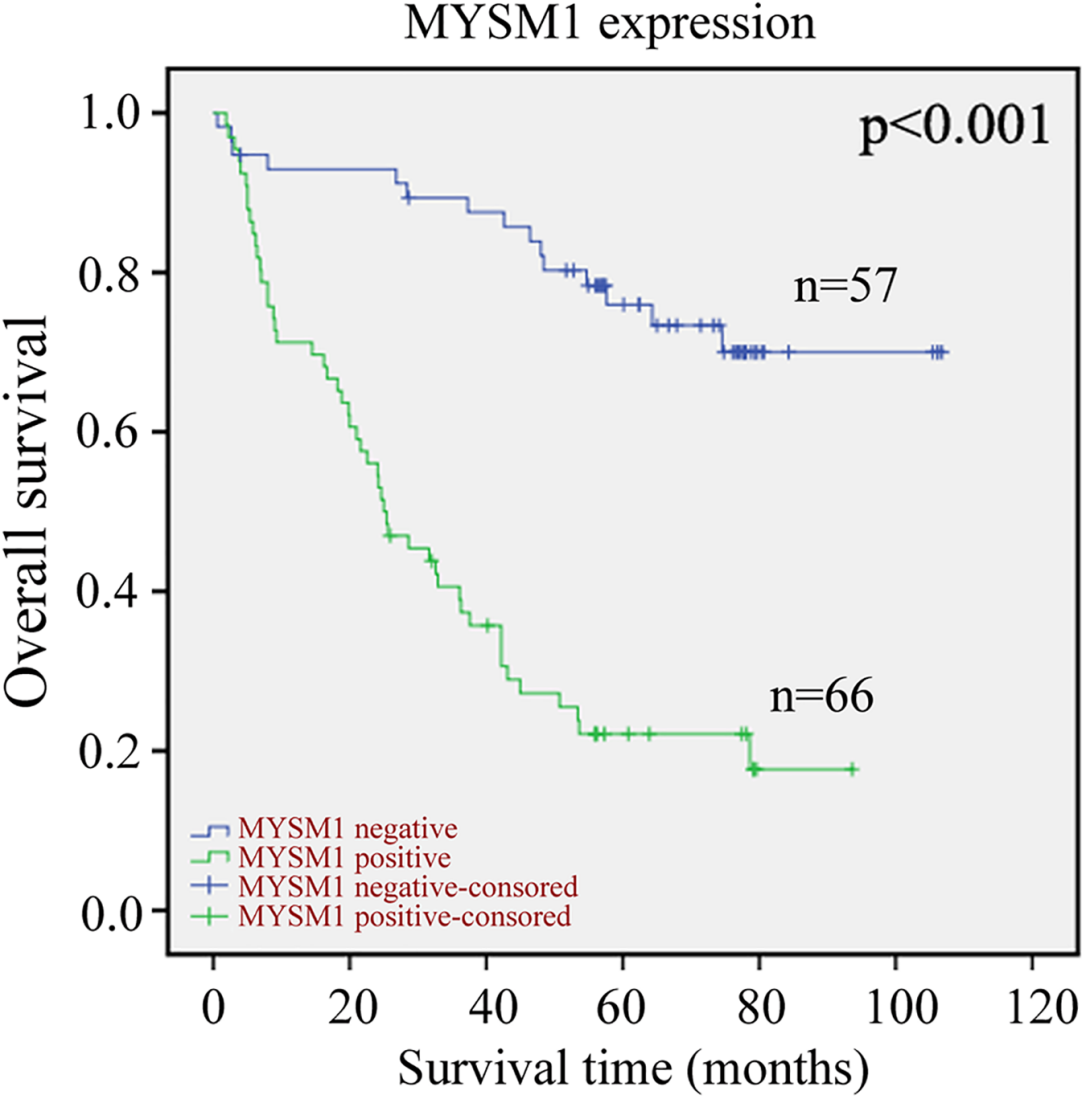
Kaplan-Meier survival analysis in patients with CRC. Overall survival curves for patients according to the negative (n = 57) and positive (n = 66) expression levels of MYSM1 of immunohistochemical variables in tumor cells.

The Kaplan-Meier curves with a log-rank test indicated that patients with positive MYSM1 expression seemed to be remarkably correlated with poor overall survival than those CRC patients with negative MYSM1 expression (P<0.001, Fig 4).Thisresultsuggestedthat the MYSM1 expression in tumor cells was an promising adverse predictive factor for overall survival of CRC patients.Furthermore, we determined that the expression of MYSM1 in tumor cells wasan independent predictorof OS according to the multivariate Coxmodel analysis (Table 2).

**Table 2:** Multivariate analyses of factors associated with OS.

Survival	Category	HR (95%CI)	p-value
**AJCC stage**	I-II vs III-IV	1.984 (1.192–3.300)	<0.001*
**T stage**	T1-3 vs T4	1.843 (1.043–3.258)	0.002*
**MYSM1**	Negative vs Posive	1.936 (1.388–2.706)	<0.001*

^*^P < 0.05, statistically significant.

### MYSM1 silencing decreases migration and invasion in CRC SW480 cells

**Fig 5:**
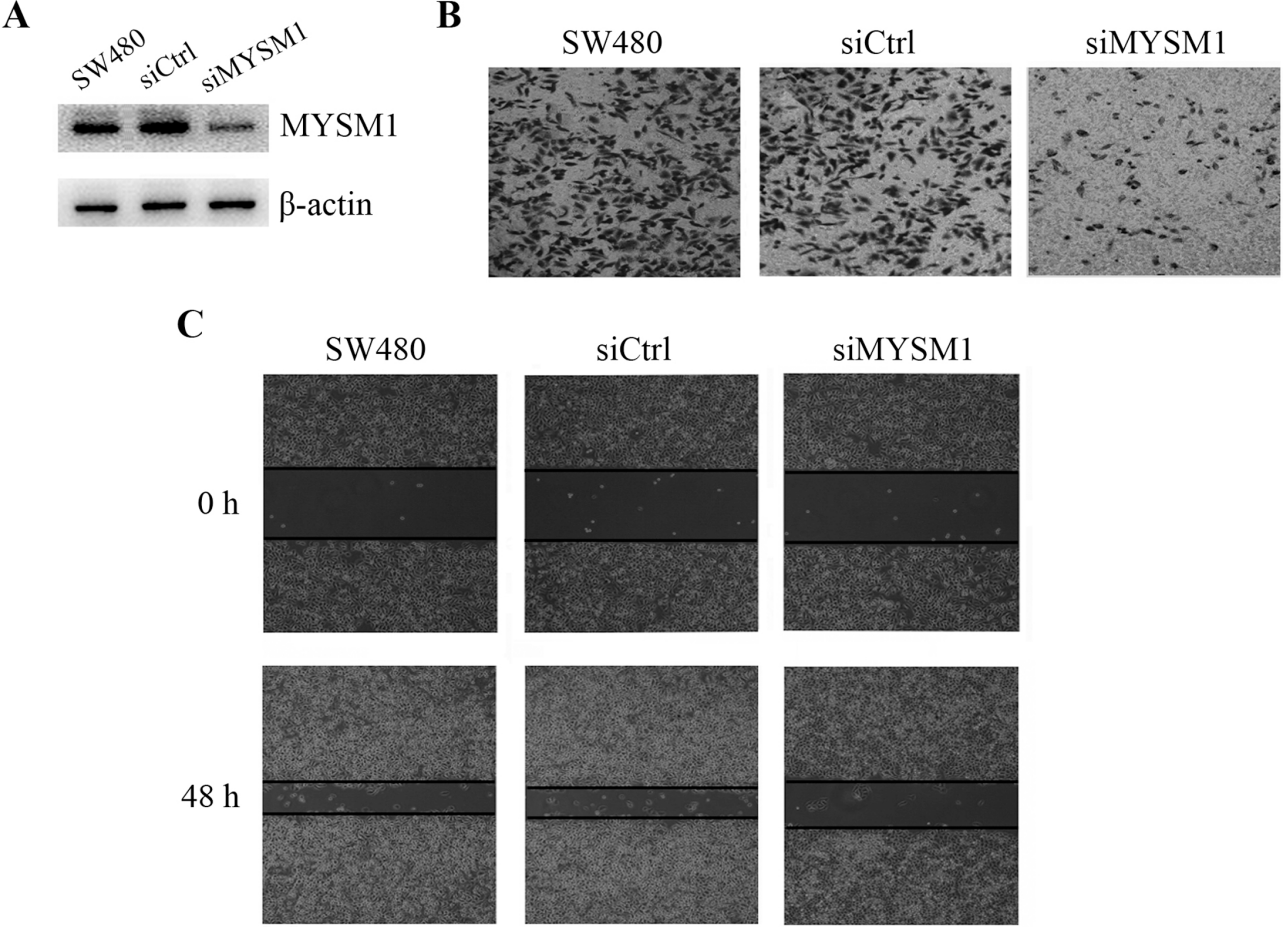
MYSM1 silencing decreases migration and invasion in CRC SW480 cells. (A) CRC SW480 cells were transfected with or without siRNA against MYSM1 (siMYSM1) or control siRNA (siCtrl). Western blot analysis was used to detect the interference efficiency of MYSM1 protein in cells.MYSM1 protein was examined using the corresponding antibody.β-actin was detected as internal reference.(B) The invasion abilities of MYSM1-silenced and non-silenced cells were evaluated by Transwell assay. (C)The migration abilities of MYSM1-silenced and non-silenced cells were evaluated by Scratch assay.

To verify the role of MYSM1 in the metastasis of CRC, we knocked down *MYSM1* expression by siRNA technology and detected the migration and invasion abilities in CRC SW480 cells. Western blot analysis showed that *MYSM1* siRNA significantly reduced the expression of MYSM1 protein compared with siRNA non-transfected or control siRNA transfected SW480 cells (Fig 5A).Transwell assay and Scratch assay indicated that *MYSM1* silencing markedly inhibited cellular invasion and migration in SW480 cells (Fig 5B and 5C). These data demonstrated that MYSM1 played an important role in CRC metastasis and was positively associated with celluar motility in SW480 cells.

## Discussion

Protein ubiquitination plays an important role in multiple cell biological processes, including DNA repair, gene transcription, protein degradation and trafficking, and cell cycle modulation [13]. Ubiquitination on H2A is usually correlated with gene silencing and X chromosome inactivation [14]. H2A deubiquitination is mediated by a series of deubiquitinases.MYSM1, as H2A deubiquitinase, contains Myb-like, SWIRM, and MPN domain that hydrolyze the isopeptide bonds of ubiquitin chains by an intrinsic metalloprotease activity [15]. MYSM1 regulates transcription by a regulatory complex coordinating histone acetylation, H2A deubiquitination, and linker histone H1 disassociation from the nucleosome [1]. Recently, most studies mainly focus on the regulation of MYSM1 on hematopoietic stem cell (HSC) function, hematopoietic progenitor survival, and lymphocyte development [3,5,16]. However, the physiological functions for MYSM1in cancer remain unclear. In the present study, we investigated the correlation between MYSM2 expression in 123 CRC patients and cancer progression to determine its predictive effect inCRC.

MYSM1 is required for the activation of several target genes in prostate cancer cells and the levels of monoubiquitinylated H2A are dramatically decreased in prostate tumors [1], suggesting MYSM1 expression may be involved in tumor biological functions. Additionally, H2A deubiquitinase USP22 was reported to promote tumor progression and induces epithelial-mesenchymal transition in lung adenocarcinoma [17]. And USP22was shown to be highly expressed in colorectal cancerand expressed higher in liver metastastic tissues than primary tumor tissues [18,19]. MYSM1, as a member of H2A deubiquitinase family, might play a similar role in cancer progression. In this study, we detected MYSM1expression in tumor specimens resected from 123 CRC patients by immunochemistry and Western blot analysis.Theresults showed MYSM1was significantly highly expressed in primary carcinoma tissues compared with normal mucosa tissues, and MYSM1was significantly increased in liver metastasis tissues compared with primary carcinoma tissues, suggesting that high MYSM1 expression was positively correlated with CRC tumorigenesis and development. In addition, association analyses by Pearson’s chi-square test verified that MYSM1 in tumors was positively correlated with tumor status and clinical stage. However, MYSM1 was not associated with tumor size of CRC patients possibly resulting from MYSM1 did not affect cellular growth rate (data not shown).Metastasis, the final step in the progression of many solid tumors, is one of the most fatal factors of cancer. CRC exhibits the rather high mortality in all of the malignancies due to its high metastasis, commonly invading or spreading to other parts of body [20].Celluar migration and invasion abilities represent the metastatic ability of tumor cells. In the present study, we knocked down MYSM1 expression by transfecting siRNA for MYSM1 to CRC SW480 cells and found that MYSM1 silencing inhibited the migration and invasion of CRC cells, indicating that MYSM1 expression was positively associated with cell migration and invasion, which was identical to the clinicopathologic characteristics. These data exhibited the correlation between MYSM1 and CRC malignancy involving cell metastasis ability but not cell proliferation ability.However, it has been reported that MYSM1 suppressed cellular migration and invasion in renal carcinoma by inhibiting epithelial-mesenchymal transition [21]. These results implied that MYSM1 might play diverse roles in different types of tumor cells. Whether MYSM1 affected CRC malignancy by inducing epithelial-mesenchymal transition remains to be established. Importantly, MYSM1 expression predicted worse poor survival and clinical outcomes of CRC patients, and we found that the expression of MYSM1 in CRC cells was an independent predictor of OS.

In conclusion, we identified the expression of MYSM1 in 123 patients with CRC, and determined the correlations between clinicopathologic features and cell metastasis. Moreover, we confirmed that the protein independently predicted poor survival and prognosis. Although further studies are needed to clarify the precise mechanism of oncogenic effect of MYSM1 in CRC, understating the oncogenic role of MYSM1 may provide the basic knowledge required for the development of potential prognostic bimarkers as targeted therapy for CRC.
