# Correction: Expression of MYSM1 is associated with tumor progression in colorectal cancer

**DOI:** 10.1371/journal.pone.0186530

**Published:** 2017-10-11

**Authors:** Yongmin Li, Jingwen Li, He Liu, Yanlong Liu, Binbin Cui

There is an error in the affiliation for Yongmin Li, Jingwen Li, He Liu, Yanlong Liu, and Binbin Cui. The affiliation should be: Department of Colorectal Surgery, Harbin Medical University Cancer Hospital, Harbin, China.

There are omissions in the Funding section. The correct funding information is as follows:

This work was supported by the National Natural Science Foundation of China (NSFC, Grant No. 1272704 and 81402367), the Science&Technology Bureau of Harbin (No. 2014RFQGJ and 2015RAXYJ063) and Research Fund for the Doctoral Program of Higher Education (SRFDP, No. 20132307120012), Heilongjiang postdoctoral fund (No. LBH-Z12155) and Harbin Municipal Science and Technology Committee of Harbin outstanding academic leaders plan (No. 2015RAXYJ063). The funders had no role in study design, data collection and analysis, decision to publish, or preparation of the manuscript.
